# Effects of 2,4-Dichlorophenoxyacetic Acid on Cucumber Fruit Development and Metabolism

**DOI:** 10.3390/ijms20051126

**Published:** 2019-03-05

**Authors:** Chaoyang Hu, Huiyu Zhao, Jianxin Shi, Jian Li, Xiangbo Nie, Guiling Yang

**Affiliations:** 1 Key Laboratory of Marine Biotechnology of Zhejiang Province, Key Laboratory of Applied Marine Biotechnology of Department of Education, School of Marine Sciences, Ningbo University, Ningbo 315211, China; huchaoyang@nbu.edu.cn; 2 Lab (Hangzhou) for Risk Assessment of Agricultural Products of Ministry of Agriculture, Institute of Quality and Standard for Agricultural Products, Zhejiang Academy of Agricultural Sciences, Hangzhou 310021, China; zhaohuiyu64@163.com (H.Z.); 13003619086@163.com (J.L.); 3 Joint International Research Laboratory of Metabolic & Developmental Sciences, School of Life Sciences and Biotechnology, Shanghai Jiao Tong University, Shanghai 200240, China; jianxin.shi@sjtu.edu.cn; 4 Shaoxing Jin Shuo Agricultural Technology Co., Ltd., Shaoxing 312000, China; xbnie@126.com

**Keywords:** *Cucumis sativus* L., 2,4-D, fruit, amino acids, flavonoids, TCA cycle, PCA, OPLS-DA, metabolomics, nutrition

## Abstract

The auxin-like compound 2,4-dichlorophenoxyacetic acid (2,4-D) has been widely used as a plant growth regulator in cucumber fruit production; however, its influence on fruit development and metabolism has not been evaluated. In this study, the phenotype of cucumber fruits in both 2,4-D treatment and non-treatment control groups were recorded, and the metabolome of different segments of cucumber fruit at various sampling time points were profiled by a standardized non-targeted metabolomics method based on UPLC-qTOF-MS. The application of 2,4-D increased the early growth rate of the fruit length but had no significant effect on the final fruit length, and produced cucumber fruits with fresh flowers at the top. The 2,4-D treatment also affected the cucumber fruit metabolome, causing significant changes in the stylar end at 4 days after flowering (DAF). The significantly changed metabolites were mainly involved in methionine metabolism, the citric acid cycle and flavonoid metabolism pathways. At the harvest stage, 2,4–D treatment significantly decreased the levels of flavonoids and cinnamic acid derivatives while increased the levels of some of the amino acids. In summary, exogenous application of 2,4-D can greatly alter the phenotype and metabolism of cucumber fruit. These findings will assist in exploring the mechanisms of how 2,4-D treatment changes the fruit phenotype and evaluating the influence of 2,4-D treatment on the nutritional qualities of cucumber fruit.

## 1. Introduction

The growth and development of crops are controlled by genotypes, growth environments and cultivating practices including the exogenous application of plant growth regulators (PGR) [1]. PGRs, both natural and synthetic, have been widely used in horticulture to manipulate plant growth and development to meet market demands [1]. There are more than 20 types of PGRs belonging to five major classes that include auxins, gibberellins, cytokinins, ethylene and abscisic acid. These compounds are active at all plant growth stages from vegetative to reproductive growth. They are used to enhance fruit color, suppress plant overgrowth, promote flower differentiation, protect flowers and fruit, accelerate fruit ripening and promote the production of seedless fruits [2]. The application of PGRs can also be used to increase yields and make crops available for market during the off season.

Despite of the advantages mentioned above, undesirable side effects are sometimes associated with PGR applications. For example, the size and mass of grapes can be increased by spraying with gibberellic acid or *N*-(2-chloro-4-pyridyl)-*N*ʹ-phenylurea (CPPU); however, this practice delays fruit ripening and maturation, as well as decreases the fruit anthocyanin content [3,4]. CPPU application to female cucumber flowers results in parthenocarpic development but decreases the levels of phenolic acid and vitamin C [5]. Therefore, the effects of PGR on crop qualities must be determined for each new application.

2,4-Dichlorophenoxyacetic acid (2,4-D) is an auxin-like compound that has been used since the 1940s as a PGR to maintain citrus fruit quality [1]. Auxin in higher plants has profound effects on plant growth and development [6,7]. Auxin displays morphogenic properties since the dynamic changes in its level, perception and signal transduction are modulated by the environment [8,9]. The application of 2,4-D at flowering and fruit setting stages can prevent flower and fruit abscission, and promote fruit growth [10,11,12,13]. 2,4-D application at the enlargement or early maturation stages can increase fruit size, prevent preharvest fruit-dropping and extend the storage time period [10,14,15]. 2,4-D has been widely used on cucumber in China since 2007 [16] for two major purposes: (1) to produce cucumber fruits with a flesh flower on top, making the cucumbers more attractive to customers; (2) to promote the fruit setting rate and the fruit growth of parthenocarpic cucumber cultivars in greenhouses where bees are excluded. In one of our previous studies, we detected residual 2,4-D in 1674 batches of vegetables (including cucumber), fruits and crops from grocery stores and evaluated the 2,4-D dietary exposure risk in China [17]. The results indicated that the 2,4-D levels in both acute and chronic dietary exposures were significantly lower than the acceptable reference dose, implying that the risk of dietary exposure to 2,4-D was quite low, not reaching a health concern level [17]. A water rinsing of 2,4-D sodium treated fruits for one minute can remove half of the 2,4-D sodium residue [17]. However, there is little information regarding the influences of 2,4-D treatment on development and metabolism of cucumber fruits.

In the present study, we examined the metabolic profiles of different segments of cucumber fruits at various time points by a non-targeted metabolomics approach. The metabolic differences between 2,4-D treated and non-treated fruits were analyzed by several statistical methods, which revealed the effects of 2,4-D on cucumber fruit development and its metabolic profile.

## 2. Results

### 2.1. Effects of 2,4-D on Cucumber Fruit Development

**Figure 1:**
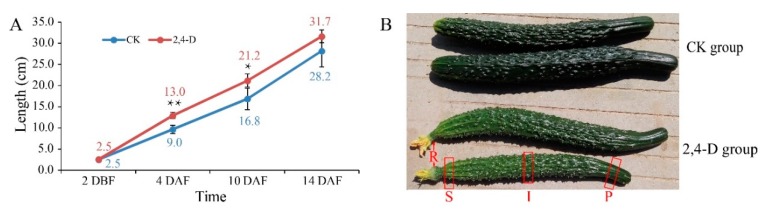
The effect of 2,4-dichlorophenoxyacetic acid (2,4-D) on the phenotype of cucumber fruit. (**A**) Cucumber fruit length over time. The number in the graph is the mean length of cucumber fruit of four replicates. Student’s *t*-test was used to determine the significance of the differential length of cucumber fruit between the 2,4-D group and the CK group at each time point. *, significant difference 0.01 < *p* < 0.05; **, extremely significant, *p* < 0.01. DBF, days before flowering; DAF, days after flowering. (**B**) Cucumber appearance at the harvest stage (14 DAF). S, stylar end; I, intermediate segment; P, peduncular end; R, expanded receptacle.

Jinchun 1, a parthenocarpic cucumber cultivar (2*n* = 14), was grown in a greenhouse in Hangzhou. Whole cucumber ovary (about 2.5 cm long), at about 2 days before flowering (2 DBF), was dipped with 2,4-D sodium solution at 100 mg/L (2,4-D group) or pure water (CK group) for 1–2 s according to the farmers’ practices, and the remainder of the ovaries or cucumber fruit on the same plant were removed. At 4 and 10 days after flowering (DAF), the lengths of cucumbers from the 2,4-D treatment group were significantly longer than those in the CK group (Figure 1A). However, at harvest (14 DAF), there was no significant difference in fruit lengths between the treatment and non-treatment groups (Figure 1A). The result indicated that 2,4-D increased the early growth rate of the fruit length but had no significant effect on the final fruit length. The receptacles of the treated cucumbers became inflated from 4 DAF to harvest (14 DAF) (Figure 1B). At harvest, the flowers at the top of the fruits appeared fresh in the 2,4-D group, but withered in the CK group; the skins of the cucumbers in the 2,4-D group were also lighter than those in the CK group (Figure 1B). These results indicated that exogenous application of 2,4-D could alter the phenotypes of cucumber fruits.

### 2.2. Kinetic Metabolic Change Patterns of Cucumber Fruit Were Similar between 2,4-D and CK Groups

In one of our previous studies, we revealed that the metabolic profiles of untreated cucumber fruits were tissue- and developmental stage-dependent [10]. In this study, we further examined the metabolic differences between different portions of the cucumber fruits in 2,4-D and CK groups at different time points (4, 10 and 14 DAF) in different portions of cucumber fruits. To minimize process errors during the experiment and to assure the comparative study of metabolic differences between the two groups is meaningful, cucumbers from the 2,4-D and CK groups were collected at the same time and analyzed together using the same mass spectrometer.

**Figure 2:**
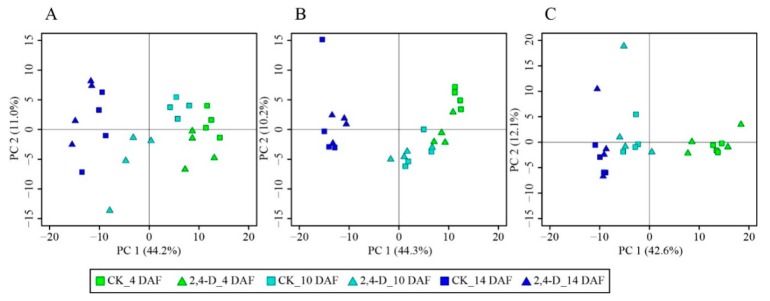
Principle component analysis of the metabolic profiles of different parts of developing cucumber fruit. Four biologycal replicates of each sample group were analyzed. Green, turquoise and blue colors represent samples at 4 DAF, 10 DAF and 14 DAF, respectively. (**A**) Principle component analysis (PCA) score plot of the stylar end of cucumber fruit. (**B**) PCA score plot of the intermediate segment. (**C**) PCA score plot of the peduncular end. Boxes and triangles denote samples from the CK and 2,4-D groups, respectively.

We identified 240 metabolites including amino acids, carbohydrates, cofactors, nucleotides, lipids, flavonoids and hydroxycinnamate derivatives (Table S1). To get a broader perspective of whether the kinetic change patterns of metabolism in cucumber fruits were affected by 2,4-D, a principle component analysis (PCA) was applied to the data to compare cucumber metabolic profiles over time and at different fruit positions. The first principal component (PC 1) accounted for more than 40% of the total variance and reflected the time course of cucumber development (Figure 2). Interestingly, the metabolic change patterns were similar between 2,4-D and CK groups, i.e., all samples in both groups shifted from the right to the left in the PCA score plots (Figure 2). At the stylar end, the 2,4-D groups were clearly separated from CK groups at all three time points (Figure 2A), indicating that the metabolome at the stylar end was significantly affected by 2,4-D treatment. In the intermediate part of cucumber fruits, samples in the 2,4-D and CK groups were clearly separated only at 4 DAF (Figure 2B), indicating that 2,4-D treatment had significant effects on the metabolic profile in the intermediate segment of cucumber fruits at the early development stage. In contrast, samples from the peduncular end of cucumber fruits in both 2,4-D treatment and CK group were all grouped together in each time point (Figure 2C), indicating that 2,4-D treatment had the least effects on the metabolic profile of cucumber fruits at the peduncular end.

### 2.3. Metabolic Pathways Affected by 2,4-D at 4 DAF

**Figure 3:**
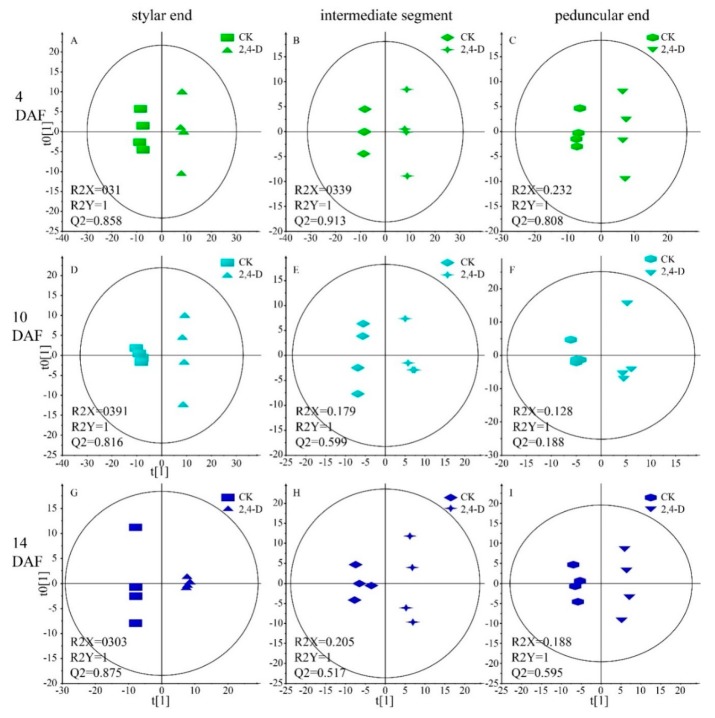
Orthogonal partial least squares projection to latent structures-discriminant analysis (OPLS-DA) score plots discriminating samples from the 2,4-D and CK group. Four biologycal replicates of each sample group were analyzed. (**A**–**C**) Score plots of the stylar, the intermediate segment and the peduncular end, respectively, at 4 DAF; (**D**–**F**) score plots of the stylar, the intermediate segment and the peduncular end, respectively, at 10 DAF; (**G**–**I**) score plots of the stylar, the intermediate segment and the peduncular end, respectively, at 14 DAF.

To obtain maximal covariance between the metabolite levels and the response variable, an orthogonal partial least squares projection to latent structures-discriminant analysis (OPLS-DA) was applied. OPLS-DA score plots showed clear separation between the 2,4-D and CK groups at 4 DAF, with satisfactory modeling and predictive abilities (R2X = 0.31, R2Y = 1, Q2(cum) = 0.858 for the stylar end, R2X = 0.339, R2Y = 1, Q2(cum) = 0.913 for the intermediate segment, and R2X = 0.232, R2Y = 1, Q2(cum) = 0.808 for the peduncular end, respectively) (Figure 3A–C). R2Y and Q2(cum) values higher than 0.5 were considered to be satisfactory in explanatory and predictive capabilities [18].

Two criteria were employed to analyze which metabolites were responsible for the discrimination of the metabolic profiles between the 2,4-D and CK groups at 4 DAF. Firstly, the variable importance in projection (VIP) values from a seven-fold cross-validated OPLS-DA model were higher than 1, since the influence of these variables on the explanation of the Y matrix was above average [18]. Secondly, the metabolite levels should be significantly different between the 2,4-D and CK groups in the Student’s *t*-test (*p* < 0.05). By these criteria, a total of 75, 74 and 34 metabolites were found to be important in the discrimination of the metabolic profiles between 2,4-D and CK group at the stylar end, the intermediate segment and the peduncular end, respectively. These metabolites were mainly enriched in the following metabolic pathways.

**Figure 4:**
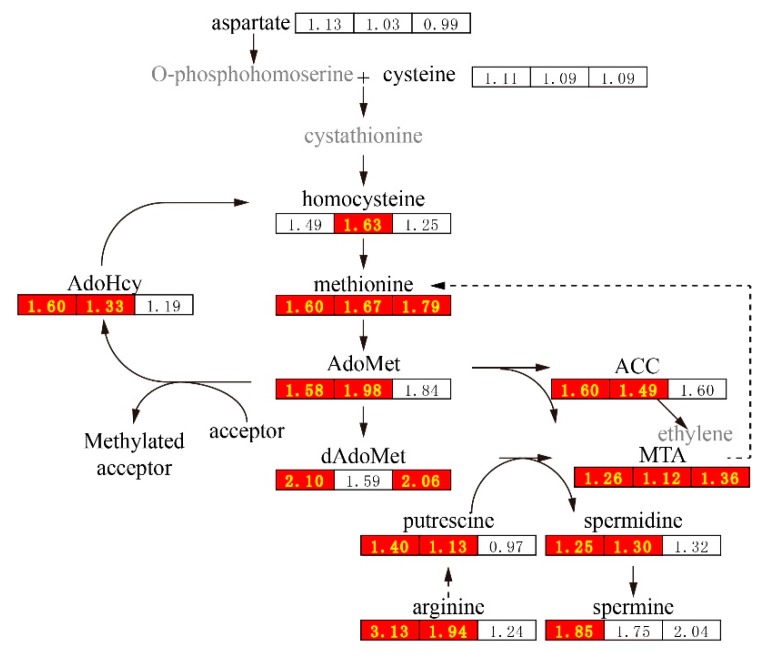
Methionine biosynthesis and metabolism pathway and the interlocked polyamine metabolism pathway that were up-regulated by 2,4-D at 4 DAF. AdoHcy, S-adenosylhomocysteine; AdoMet, S-adenosylmethionine; dAdoMet, S-adenosylmethioni- namine; MTA, 5-methylthioadenosine; ACC, 1-aminocyclopropane-1-carboxylate. The numbers in the cells are the ratios of fold changes (2,4-D group/CK group) using the average values of each group. The numbers from the left to the right are the ratios in the stylar end, the intermediate segment and the peduncular end, respectively. Red background indicates that metabolite levels was significantly higher in the 2,4-D group compared with the CK group.

Firstly, methionine biosynthesis and metabolism was distinctly altered by 2,4-D treatment (Figure 4). Methionine levels increased significantly in all three parts of cucumber fruits after 2,4-D treatment at 4 DAF. The levels of homocysteine (the terminal step substrate) and serine (the methyl group donor) for methionine biosynthesis were both significantly increased in the intermediate segment. In contrast, the levels of aspartate (carbon backbone) and cysteine (sulfur donor) were not significantly changed. The results suggest that the terminal step for methionine biosynthesis was up-regulated by 2,4-D at 4 DAF.

Methionine occupies a central position in cellular metabolism, protein synthesis and methyl-group transfer via S-adenosylmethionine (AdoMet) and it also affects polyamine and ethylene syntheses [19]. About 80% of methionine is incorporated into AdoMet [20]. We found that the AdoMet levels were significantly increased in the stylar end and the intermediate segment by 2,4-D treatment. AdoMet is primarily (90%) used for transmethylation reactions in which methionine acts as a methyl group donor for acceptors including phosphatidylcholine (PC), DNA and RNA intermediates [19,21,22]. AdoMet is also an S-adenosylhomocysteine precursor. We found that the AdoMet levels were significantly increased by 60% in the stylar end and 33% in the intermediate segment by 2,4-D treatment, but were not significantly changed in the peduncular end (Table S1). This result indicates that transmethylation reactions were enhanced in the stylar end and intermediate segment of cucumber fruits treated with 2,4-D. In the nine phosphatidylcholines (PC) detected, only the level of 2-LysoPC(18:3) in the stylar end and those of 2-LysoPC(16:0) and 2-LysoPC(18:2) in the intermediate segment were significantly decreased by 2,4-D treatment. This suggested that PC methylation levels were similar between the 2,4-D and CK groups but that the methyl transfer to other acceptors might be elevated in the 2,4-D group.

5-methylthioadenosine (MTA) can be produced from AdoMet via the ethylene or polyamine biosynthesis pathways, and converted into methionine by a series of metabolic reactions (Figure 3) [19]. The increased levels of S-adenosylmethioninamine (dAdoMet), MTA, spermidine and 1-aminocyclopropane-1-carboxylate (ACC, ethylene precursor) in 2,4-D treated groups indicated that the elevated level of MTA was from both ethylene and polyamine biosynthesis pathways. Positively charged polyamines can bind macromolecules such as DNA, RNA and proteins. They are involved in diverse processes in plants, including morphogenesis, growth and organ development [23,24]. The increased levels of three major plant polyamines (putrescine, spermidine and spermine) and the level of arginine (the precursor for polyamine biosynthesis) in the stylar and intermediate regions indicated the promotion effect of 2,4-D treatment on polyamine biosynthesis. These metabolic changes may be associated with the increased growth rate and the expanded receptacles observed in the 2,4-D group.

Secondly, the tricarboxylic acid (TCA) cycle was up-regulated by 2,4-D treatment (Table S1). The levels of succinate, α-ketoglutarate and succinate were significantly elevated in all three fruit regions. The levels of fumarate and malate were significantly increased in the stylar end and intermediate segment. The TCA cycle is responsible for ATP generation through oxidation of acetyl-CoA derived from carbohydrates, lipids and amino acids. It also provides several amino acid precursors and generates reduced NADH used in many biochemical processes [25,26]. We deduced that the elevation of the TCA cycle intermediates is likely correlated with the higher growth rate of the fruit length in the 2,4-D group at 4 DAF. Consistent with this deduction was the increased level of nicotinate (vitamin B3) in the stylar end and the intermediate segment, because nicotinate serves as the direct precursors for NAD^+^ and NADP^+^ cofactors that play essential roles in energy metabolism, including the TCA cycle [27].

In addition to the TCA intermediates, propionic acid and 3-oxoglutaric acid levels were also significantly increased in all three sampling regions. Glucosaminic acid and glucosamine levels in the stylar and intermediate segments were also elevated, although their functions in plant metabolism are still obscure.

**Figure 5:**
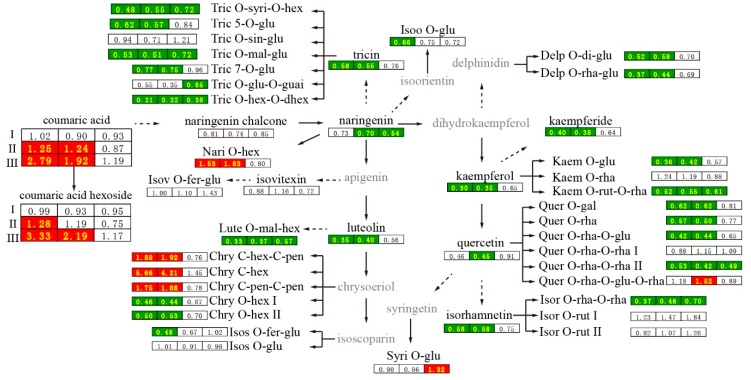
Flavonoid metabolism was altered with 2,4-D at 4 DAF. The numbers in the cells are the ratios of fold changes (2,4-D group/CK group) from the average values of each group. The numbers from the left to the right are the ratios in the stylar end, the intermediate segment and the peduncular end, respectively. Red and green background indicate the metabolite level was significantly higher and lower in the 2,4-D group compared with that in CK group, respectively. Refer to Table S3 for complete metabolite names.

Thirdly, flavonoid biosynthesis and metabolism was significantly affected by 2,4-D treatment (Figure 5). Flavonoids are ubiquitous in the plant kingdom and have many diverse functions including UV protection, insect defense, flower and fruit coloring and auxin transport inhibition [28,29]. The levels of seven flavonoids, such as isorhamnetin-*O*-rhamnoside-*O*-rhamnoside and kaempferol-3-*O*-rutinoside-7-*O*-rhamnoside, were significantly decreased in all three sampling areas at 4 DAF. The levels of 15 other flavonoids including tricin and kaempferol were significantly decreased, while the levels of naringenin *O*-hexoside and three chrysoeriol C-glycoside isomers were significantly increased in the stylar end and the intermediate segment. The levels of two isomers of the naringenin chalcone biosynthetic precursor coumaric acid and coumaric acid hexosides II and III were significantly increased in the stylar end and the intermediate segment in the 2,4-D group (Figure 4 and Table S1). These results indicated that 2,4-D treatment affected flavonoid biosynthesis in the stylar end and the intermediate segment but not in the peduncular end of cucumber fruits at 4 DAF.

### 2.4. Metabolic Differences Generated by 2,4-D at 10 DAF

We generated an OPLS-DA model with one predictive component and one orthogonal component to discriminate metabolic profiles between the 2,4-D and CK groups at 10 DAF. Score plots of samples from the stylar end and the intermediate segment showed clear separations between these two groups, with satisfactory modeling and predictive abilities (R2X = 0.391, R2Y = 1, Q2(cum) = 0.816 for stylar end, R2X = 0.179, R2Y = 1, Q2(cum) = 0.599 for intermediate segment, respectively) (Figure 3D–F). 2,4-D and CK group samples of the peduncular ends were also distinctly separated in OPLS-DA score plots with high modeling ability (R2Y = 1). However, the predictive ability of this model was very poor (Q2(cum) = 0.188) indicating that metabolic differences between the 2,4-D and CK group in the peduncular end were relatively low (Figure 3D–F).

In the stylar end, 95 metabolites were distinct between the 2,4-D and CK groups. These include 16 amino acids, seven benzene derivatives, eight carbohydrates, 30 flavonoids, 11 hydroxycinnamate derivatives, 13 lipids, 13 nucleotides and one monoterpenol glycoconjugate (Table S1). Most of the significantly changed flavonoids and hydroxycinnamate derivatives at 10 DAF overlapped with those at 4 DAF. The significantly changed metabolites of the other classes differed from those at 4 DAF. The 30 metabolites with the highest VIP values for discrimination of the stylar end between the 2,4-D and CK groups were listed in Table 1. Among them, the levels of 16 flavonoids and seven hydroxycinnamate derivatives were all significantly decreased in the 2,4-D group. Unlike those observed at 4 DAF, the levels of most metabolites involved in methionine metabolism and TCA cycle were not significantly increased at 10 DAF with the exception of AdoMet, dAdoMet and α-ketoglutarate. In addition to the flavonoids, metabolites associated with oxidative stress were also significantly changed at 10 DAF by 2,4-D. The levels of oxidized glutathione, alanine, salicylic acid, salicylic acid glucoside and ascorbate decreased significantly. The levels of oxidized lipids (9-OxoOTrE, 13-HOTrE and 9-HOTrE) and those of the unsaturated fatty acids (stearidonic, punicic acid and γ-aminobutyrate) were increased at 10 DAF, l, indicating the oxidative status in the stylar end were altered at 10 DAF by 2,4-D (Table S1).

In the intermediate segment, 33 metabolites were significantly altered, including 10 flavonoids, seven hydroxycinnamate derivatives, seven amino acids and four benzene derivatives (Table S1). The five metabolites with the highest VIP values were coumaric acid hexoside III, 2-hydroxybenzaldehyde, chrysoeriol C-hexoside, coumaric acid III and isoscoparin-2′-*O*-glucoside. In the peduncular end, the levels of six metabolites were significantly changed. The levels of dAdoMet, *N*-acetylglutamate and 2-LysoPC (16:0) were significantly increased while those of valine, histidine and pipecolic acid were decreased.

### 2.5. Metabolic Difference between the 2,4-D and CK Groups at the Harvest Stage

An OPLS-DA model was generated with one predictive component and one orthogonal component to discriminate between the 2,4-D group and CK groups at 14 DAF. Score plots of samples from all three regions of cucumber fruits showed clear separations between groups (Figure 3G–I). The modeling and predictive abilities were satisfactory (R2X = 0.303, R2Y = 1, Q2(cum) = 0.875 for the stylar end, R2X = 0.205, R2Y = 1, Q2(cum) = 0.517 for the intermediate segment and R2X = 0.188, R2Y = 1, Q2(cum) = 0.595 for the peduncular end, respectively). Using the criteria mentioned above, we identified 66, 21 and 19 metabolites responsible for the discrimination of the 2,4-D group from CK group in the stylar end, the intermediate segment and the peduncular end, respectively.

The levels of 26, 12 and four amino acids were significantly changed in the stylar end, in the intermediate segment, and in the peduncular end, respectively (Table S1). This result indicates that amino acid metabolism was strongly affected by 2,4-D in the stylar end while only slightly affected in the peduncular end. In addition, the patterns of amino acid changes in different cucumber fruit regions were also different. The levels of nine amino acids, including arginine, argininosuccinate and three essential amino acids isoleucine, leucine and tryptophan were significantly increased in the stylar end. The levels of AdoMet and metabolites involved in polyamine metabolism, including spermine, spermidine and putrescine, were only significantly decreased in the stylar end. The levels of the human essential amino acids methionine, phenylalanine, histidine and lysine, as well as tyrosine, were significantly increased in the stylar end, but decreased in the intermediate segment. The levels of another essential amino acid (valine) as well as an intermediate metabolite in lysine degradation (2-aminoadipic acid) were significantly decreased in both the stylar and peduncular ends (Table S1).

The levels of 17 flavonoids including glycosylated forms of chrysoeriol, kaempferol, quercetin and tricin were significantly decreased in the stylar end. The numbers of the significantly changed flavonoids in the intermediate segment and the peduncular end were only one and three, respectively. This indicated that 2,4-D had a negative effect on the accumulation of nutritional flavonoids in the stylar end but a negligible effect in other regions at 14 DAF. The levels of eight hydroxycinnamate derivatives were significantly changed by 2,4-D in the stylar end (Table S1). Seven of these eight hydroxycinnamate derivatives (including two coumaric acid isomers, two coumaric acid hexoside isomers and coumaroyl quinic acid) were significantly decreased in the stylar end. The level of quinate was significantly increased by 2,4-D in both the intermediate segment and the peduncular end.

The levels of four carbohydrates such as glucose were significantly decreased, while norophthalmate and α-ketoglutaramate were increased in the stylar end (Table S1). The levels of aconitic and glucosaminic acids were simultaneously decreased in the intermediate segment and the peduncular end. For lipids, the levels of sphinganine, phytosphingosine, 2-hydroxyadipate and myristic acid decreased in the stylar end while those of 2-LysoPC(18:3) and glycero-3-PC increased in the intermediate end. In the peduncular end, the levels of 3-hydroxy-3-methyl-glutaric acid, 2-LysoPC(18:3) and linolenoyl ethanolamide were increased.

## 3. Discussion

Different plant growth regulators may have different effects on the same crop, thus, each one needs to be evaluated individually. For example, female cucumber flowers treated with 100 mg/L CPPU, naphthaleneacetic acid (NAA) or gibberellin A_4_ + A_7_ (GA_4 + 7_) can all result in parthenocarpic fruits, however, the qualities of the fruits after storage are different [5]. Phenolic acid and vitamin C contents decrease after CPPU treatment; the total flavonoids and protein content increase after GA_4+7_ treatment; and nutritional characteristics in cucumber fruits are not affected by NAA treatment [5]. In the current study, we investigated the effects of 2,4-D on the morphology and metabolism of cucumbers in different regions at various time points.

The cucumber fruit morphology was significantly changed by 2,4-D, i.e., the receptacles were inflated and the flowers were still fresh at the harvest. When applied to the post-harvest citrus fruit in a dip treatment, sodium salt of 2,4-D at 500 mg/L can cause retarded calyx abscission [30]. The application of 10 mg/L 2,4-D significantly decrease fruit drop of navel orange (*Citrus sinensis* L.) and carambola (*Averrhoa carambola* L.) by enhancing retention [31,32]. 2,4-D may delay cucumber flower senescence by altering the levels of many endogenous hormones as revealed in transcriptomic and proteomic studies of post-harvest citrus fruits [33]. Application of 2,4-D resulted in a dramatic increase in ethylene level in barley, soybean, cotton and grain sorghum [34,35], increased abscisic acid level in *Cucurbita pepo* [36] and a significant alteration in auxin level in other plants [37]. The observed morphology changes in 2,4-D treated cucumber fruits could result from 2,4-D induced imbalance of endogenous plant hormones, which merits further comprehensive investigations. In the current study, we also found that 2,4-D increased the early growth rate of the cucumber fruit length, which is consistent with the reported study that 2,4-D can promote cell division of tobacco cells [38].

The largest metabolic changes between the 2,4-D and the CK groups was observed in the stylar end while the changes in the peduncular end was the smallest. There are several possible explanations for these results. Firstly, the peduncular end of a cucumber fruit is smooth, making it difficult to adsorb 2,4-D solution. The intermediate parts and the stylar end of cucumbers are both covered with trichomes which make them easy to adsorb 2,4-D solution. In addition, the flower bud is covered with trichomes that may facilitate adsorption. The stylar end can absorb 2,4-D solution from the flower bud through capillary action, so 2,4-D absorption could likely be maximal in the stylar end while minimal at the peduncular end. Secondly, the fresh flowers and the expanded receptacles may have significant impact on the metabolism of the stylar end. Thirdly, different regions of cucumbers possess specific tissue structures and metabolic requirements, making them response differently to 2,4-D treatment. The largest and smallest metabolic differences between the 2,4-D and CK groups were observed at 4 and 14 DAF, respectively. There are some deductions to explain this phenomenon. Firstly, the fruit size was bigger in the 2,4-D group than in the CK group at 4 DAF, but not significantly different at 14 DAF. It was reported that the levels of most metabolites decrease together with the fruit expanding [10,39]. Secondly, the levels of 2,4-D in cucumber fruit may be the highest at 4 DAF and lowest at 14 DAF, since 2,4-D can be degraded by cucumber [40,41]. We speculate that the effect of 2,4-D treatment on the changes of metabolic profiles between the 2,4-D and CK groups was more significant than effects due to the different fruit size at 4 DAF and 14 DAF. Since the significantly changed metabolites between the 2,4-D and CK groups at 4 DAF were concentrated in three metabolic pathways, including methionine metabolism pathway and flavonoid metabolism pathway, which is consistent with the results in other plants [42,43,44,45].

The effects of 2,4-D on many metabolic pathways are conserved among different plant species, likely through its effects on the homeostasis of other plant hormones. In Arabidopsis and wheat, exogenous 2,4-D treatment enhances ethylene biosynthesis and signaling pathways by up-regulating ACC synthase, AdoMet synthase and ACC oxidase while down-regulating negative regulators CTR1 and ERS [42,43]. 2,4-D promotes the ripening of the detached and unripe fruits, such as bananas, apples and pears [46], which is associated with the increased levels of ethylene [47]. Though we did not examine the ethylene content, the significantly increased levels of precursors for ethylene biosynthesis in the stylar end and the intermediate segment of cucumber fruit in 2, 4-D groups at 4 DAF (Figure 4) might be an indicator of ethylene accumulation in 2,4-D treated cucumber fruit. Excessive use of 2,4-D often results in the generation of reactive oxygen species (ROS) that is associated with enhanced antioxidant pathways as illustrated in 2,4-D treated mung beans and peas [44,45], mango fruit under chilling injury [48] and potato infected with *Alternaria solani* [49]. The observed significant increased levels of some oxidation products, such as oxidized lipids, and contrastingly decreased levels of antioxidants, such as ascorbate, in the stylar end of the cucumber fruit at 10 DAF, indicated that 2,4-D treatment resulted in the generation of ROS in cucumber fruit. In addition, exogenous application of salicylic acid regulates ROS level by affecting the activities of CAT, SOD and POD in plant [50,51]. The detected significantly decreased levels of salicylic acid and salicylic acid glucoside in the stylar end at 10 and 14 DAF of 2,4-D groups (Table S1) led us to speculate an elevated ROS level in 2,4-D treated cucumber fruit, especially in the stylar end. Furthermore, the observed remarkably decreased levels of metabolites associated with hydroxycinnamate metabolism, the upstream of flavonoid biosynthesis in the phenylpropanoid metabolic pathway [52], in the stylar end from 4 DAF to 14 DAF, wasconsistent with previous studies, in 2,4-D inhibits total flavonoid production in callus cultures of *Astragalus missouriensis* Nutt and carrot cells by inhibition of the activities of enzymes involved in phenylpropanoid metabolism [53,54]. Besides, 2,4-D is reported to inhibit the accumulation of anthocyanin by down-regulating of anthocyanin biosynthetic pathways genes in grape berries [55]. The expression of phenylpropanoid pathway genes, such as chalcone synthase (CHS) and cinnamate 4-hydroxylase (C4H), in wheat was induced by 2,4-D [43,56]. Because levels of flavonoids and hydroxycinnamates in the intermediate segment and peduncular ends were significantly changed by 2,4-D treatment at 4 DAF although these differences were no longer apparent at 14 DAF, we speculated that the expression of genes or the activities of enzymes involved in phenylpropanoid metabolis in cucumber fruit were also inhibited by 2,4-D. The phenylpropanoid metabolism were differentially regulated between wheat and cucumber may be due to the different sensitivities of wheat and cucumber to 2,4-D [43].

The metabolic differences between 2,4-D and CK groups at earlier time points disappeared largely by the harvest stage. However, the levels of some flavonoids and amino acids, which are important nutrients for humans, were still significantly changed by 2,4-D at 14 DAF. The levels of some amino acids, such as leucine, isoleucine and tryptophan, were increased in the stylar end, but were not significantly changed in the other two regions of cucumber fruit. We deduced that levels of these amino acids in a whole cucumber fruit were *per se* increased by 2,4-D. Levels of other amino acids, such as lysine and histidine, were increased in the stylar end, but decreased in the intermediate segment. Although we did not measure the changes of amino acid in the whole cucumber, and it is hard to judge its change after 2,4-D treatment, our metabolic data clearly showed that 2,4-D treatment had different effects on amino acids in various fruit regions. Levels of 17 flavonoids were significantly decreased in the stylar end while some of them were also significantly changed in the intermediate segment and the peduncular end after 2,4-D treatment, indicating that levels of flavonoids in a whole cucumber fruit were probably decreased by 2,4-D. Thus, we recommend to reduce the concentration of 2,4-D sodium solution while using in cucumber fruit production in order to reduce its negative effects on the nutritional quality of cucumber.

## 4. Materials and Methods

### 4.1. Materials

The seeds of cucumbers (cv. Jinchun 1) were purchased from Tianjin Ke Run Agricultural Technology Co., Ltd. (China) and were planted in a greenhouse in Hangzhou (31.03 °N, 121.45 °E), Zhejiang province, China, in September 2016. The commercial products of 85% 2,4-D sodium wet table powder were obtained from Guoguang Co., Ltd. (Sichuan, China). At 2 days before flowering (2 DBF), the whole cucumber ovaries were dipped in the sodium salt of 2,4-D at 100 mg/L (2,4-D group) or pure water (CK group) for 1–2 s. Each plant retained the only one treated cucumber ovary and the remainders were removed. Four biological replicate samples of the stylar ends, the intermediate segments and the peduncular ends of the cucumber fruit were taken at 4, 10 and 14 days after flowering (DAF) and immediately frozen in liquid nitrogen. The samples were lyophilized for 48 h and stored at −80 °C until metabolomic analysis. Samples collected from two cucumber fruits were pooled together as one biological replicate.

### 4.2. Metabolite Profiling

Samples were prepared for metabolite analysis by grounding lyophilized samples into a fine powder. Extraction of 20 mg powder per sample was initiated by adding 500 µL 80% methanol and by vortexing for 3 min. The mixture was kept at room temperature for 10 min and then centrifuged at 13,000× *g* for 10 min. The supernatant was filtered through a syringe filter (0.22 µm) and placed in a sampling vial.

Liquid chromatography analysis was performed on an Agilent 1290 Infinity II LCTM system (Agilent, Santa Clara, CA, USA). A 1.0 µL aliquot of the filtrate was injected into an Agilent Eclipse-plus C18 column (150 × 3.0 mm i.d, 1.8 μm) with a column temperature of 30 °C. The mobile phase consisted of A (0.1% formic acid in water) and B (100% acetonitrile) for both positive and negative ion mode. The column was eluted with gradient conditions of the mobile phase as follows: 0 min, 98.0% A; 1.0 min, 98% A; 5.0 min, 60% A; 12.0 min, 30% A; 15.0 min, 5% A; 20.0 min, 5% A. The flow rate was 0.40 mL/min. All the samples were kept at 4 °C during the analysis.

Mass spectrometric data was collected using an Agilent 6545 Q-TOF MS equipped with an electrospray ionization (ESI) source. The ESI source was operated in positive and negative ionization modes with a capillary voltage of 3.5 kV for both modes. The nozzle voltage was 500 V (+) and 1500 V (-), the fragmentation voltage 110 V and the nebulizer at 45 psi. The sheath gas and dry gas were both set at 8 L/min. Collision-induced dissociation (CID) voltage was applied at 10 eV, 30 eV and 50 eV and centroid data was recorded from 50 to 1100 *m*/*z* with a scan rate at 2 spectra/s.

The metabolites were annotated by searching the Personal Compound Database and Library (PCD/PCDL), which was established in our previous study [57]. MS and MS/MS of the compounds were analyzed using the Metlin [58] and the Massbank databases [59]. Data acquisition, metabolite annotation and peak area extraction were performed with MassHunter Acquisition 7.0, MassHunter Qualitative 7.0 and Mass Profinder 8.0 software (Agilent), respectively.

### 4.3. Data Analysis

The sample weight and non-normalized peak areas are available in Table S2. For data normalization, peak areas were divided by the sample weight and the median value of each metabolite. The missing values of a giving metabolite were adjusted to the detected minimum value of the same metabolite for statistical analysis, assuming that they were below the limits of instrument detection sensitivity. The final statistics matrix with normalized data are available in Table S3.

The PCA and OPLS-DA were performed with the SIMCA-P 13.0 software package using UV scaling. The Student’s *t*-test was employed to identify metabolites that differed significantly (*P*-value ≤ 0.05) between 2,4-D and CK groups using the log transformed data matrix (Table S1). The figures were edited using Adobe Illustrator CS5 software for better resolution.

## 5. Conclusions

In the present study, we investigated the metabolic profiles of three regions of cucumber fruits over time courses with and without 2,4-D treatment. Phenotypically, 2,4-D treated flowers remained closed and did not detach even at harvest at 14 DAF. In addition, the fruit receptacles became significantly inflated in the 2,4-D treatment group. The metabolic effects of 2,4-D on cucumber fruit were the greatest at 4 DAF and almost disappeared at harvest. From the stylar end to the peduncular end, the effect of 2,4-D on cucumber metabolism was gradually reduced. This study demonstrates that 2,4-D sodium could have a significant impact on the nutritional quality of cucumber, implying that both nutritional and safety effects should be considered when 2,4-D sodium is applied on cucumber production. The study will also guide future studies to understand the underlying mechanisms of 2,4-D treatment on cucumber metabolism and development.

## Figures and Tables

**Table 1:** The top 30 most important metabolites for discrimination of the stylar end of the 2,4-D group from that of the CK group at 10 DAF.

Metabolite	Class ^a^	Ratio ^b^	*p*-Value ^c^	VIP ^d^
salicylic acid	Ben	0.28	4.25 × 10^−3^	1.44
salicylic acid O-glucoside	Ben	0.25	3.50 × 10^−3^	1.39
citraconate	Car	0.65	3.76 × 10^−4^	1.54
aconitic acid	Car	0.41	3.56 × 10^−3^	1.41
ascorbate	Cof	0.04	6.72 × 10^−4^	1.54
naringenin	Fla	0.08	9.07 × 10^−4^	1.58
isoorientin 2′-*O*-glucoside	Fla	0.11	1.10 × 10^−3^	1.56
quercetin-3-*O*-rhamnosyl(1-2)-glucoside-7-*O*-rhamnoside	Fla	0.08	2.01 × 10^−3^	1.56
kaempferol-3-*O*-rhamnoside	Fla	0.02	2.55 × 10^−4^	1.54
tricin-*O*-hexoside-deoxyhexoside	Fla	0.04	1.17 × 10^−6^	1.51
isovitexin	Fla	0.03	2.03 × 10^−4^	1.49
Naringenin *O*-hexoside	Fla	0.03	1.43 × 10^−3^	1.48
kaempferol-3-*O*-glucoside	Fla	0.27	0.014	1.46
quercetin-3-*O*-rhamnoside-7-*O*-glucosid	Fla	0.28	0.013	1.45
tricin 7-*O*-(6′-*O*-malonyl)-β-d-glucopyranoside	Fla	0.31	0.015	1.44
astilbin	Fla	0.02	8.47 × 10^−4^	1.44
luteolin	Fla	0.27	0.017	1.44
chrysoeriol C-pentoside-C-pentoside	Fla	0.11	2.40 × 10^−3^	1.41
kaempferol	Fla	0.29	2.10 × 10^−3^	1.41
luteolin-O-malonylhexoside	Fla	0.29	8.71 × 10^−3^	1.41
chrysoeriol-C-hexoside-C-pentoside	Fla	0.1	2.73 × 10^−3^	1.4
caffeic acid hexoside II	Hyd	0.02	7.54 × 10^−5^	1.59
ferulic acid hexoside II	Hyd	0.08	3.75 × 10^−5^	1.53
coumaric acid II	Hyd	0.11	6.40 × 10^−4^	1.51
coumaric acid hexoside II	Hyd	0.07	7.32 × 10^−4^	1.49
feruloyl quinic acid I	Hyd	0.49	6.77 × 10^−3^	1.47
feruloyl quinic acid II	Hyd	0.28	8.36 × 10^−3^	1.46
coumaroyl quinic acid	Hyd	0.26	5.02 × 10^−3^	1.44
2-hydroxyadipate	Lip	0.56	0.013	1.43
terpenyl-pentosyl-glucoside I	Mon	0.06	1.43 × 10^−3^	1.41

^a^ Ben: Benzene derivatives; Car: Carbohydrates; Cof: Cofactors; Fla: Flavonoids; Hyd: Hydroxycinnamate derivatives; Lip: Lipids; Mon: Monoterpenol glycoconjugates. ^b^ Mean values of the 2,4-group/ CK group. ^c^ *P*-value was determined using the Students *t*-test algorithm using Excel2013 software and was deemed significant at the 5% level. ^d^ Variable importance in projection (VIP) was obtained from OPLS-DA modeling.

## Supplementary Materials

The following are available online at https://www.mdpi.com/1422-0067/20/5/1126/s1.

## Abbreviations

2,4-D	2,4-dichlorophenoxyacetic acid
ACC	1-aminocyclopropane-1-carboxylate
AdoMet	S-adenosylmethionine
CID	Collision-induced dissociation
CK	Black controls
CPPU	*N*-(2-chloro-4-pyridyl)-*N*′-phenylurea
dAdoMet	S-adenosylmethioninamine
DAF	Days after flowering
DBF	Days before flowering
ESI	Electrospray ionization
HOTrE	hydroxyl-octadecatrienoate
LysoPC	lysophosphocholine
MTA	5-methylthioadenosine
NADH	Reduced nicotinamide adenine dinucleotide
NAD^+^	Nicotinamide adenine dinucleotide
NADP^+^	Nicotinamide adenine dinucleotide phosphate
OPLS-DA	Orthogonal partial least squares projection to latent structures-discriminant analysis
PCA	Principle component analysis
PCD/PCDL	Personal compound database and library
PGR	Plant growth regulators
TCA	Tricarboxylic acid
UHPLC-qTOF-MS	Ultra-high performance liquid chromatography-quadrupole time of flight mass spectrometry
UV	Ultraviolet ray
VIP	Variable importance in projection
